# Effects of Living Mulch and Branches Mulching on Soil Moisture, Temperature and Growth of Rain-Fed Jujube Trees

**DOI:** 10.3390/plants11192654

**Published:** 2022-10-09

**Authors:** Min Tang, Xiaodong Gao, Pute Wu, Hongchen Li, Chao Zhang

**Affiliations:** 1 College of Hydraulic Science and Engineering, Yangzhou University, Yangzhou 225009, China; 2 Institute of Water-Saving Agriculture in Arid Areas of China, Northwest Agriculture and Forestry University, Yangling 712100, China; 3 School of Resources and Environmental Engineering, Ludong University, Yantai 264011, China

**Keywords:** jujube, mulching, soil moisture, soil temperature, leaf area index, stem diameter

## Abstract

The influence of different mulching measures on soil moisture, soil temperature, and crop growth was investigated during the jujube growing season in rain-fed jujube orchards using micro-plot experiments. The mulching treatments included clean tillage (CT, control treatment), jujube branches mulching (JBM), and white clover planting (WCP). The results revealed that: (1) The average soil moisture content of JBM was greater than that of CT by 3.76% and 2.34%, respectively, during the 2013 and 2014 jujube growth periods, and its soil water deficit was minimal in each soil layer from 0 to 70 cm. WCP had the greatest soil water deficit. The average soil moisture content of the 0–70 cm soil layer in WCP was 3.88% and 5.55% lower than that in CT during the 2013 and 2014 jujube growth seasons, respectively (*p* < 0.05). (2) JBM had the highest annual average soil moisture content in each soil layer from 0 to 70 cm, followed by CT, while WCP had the lowest. White clover and jujube competed for water in the 20–40 cm soil layer, and JBM had the lowest variation in soil moisture. (3) Mulching with jujube branches and planting white clover could both control the temperature of the 0–25 cm soil layer and narrow the daily temperature range, with JBM being the least affected by air temperature. (4) Jujube’s leaf area index and stem diameter increase in JBM were both significantly greater than in CT and WCP. In conclusion, using pruned jujube branches as surface mulch is appropriate for rain-fed jujube orchards because it can preserve soil moisture, regulate soil temperature, and promote jujube growth.

## 1. Introduction

Jujube (*Ziziphus jujuba* Mill.), a drought-tolerant economic forest species, is widely planted and popular in semi-arid regions and has significantly contributed to the economic growth of local agriculture and forestry, as well as the rehabilitation of the natural environment [1,2,3]. However, China’s rain-fed jujube orchards have long been dominated by clean tillage due to conventional orchard management practices and farmers’ beliefs, which has resulted in significant soil erosion, degraded soil fertility, insufficient water retention capacity, and extreme fluctuations in soil temperature of the arable layer [4,5,6]. The aforementioned occurrences not only reduce the current and potential production of jujube orchards, but they also cause a decline in yield and quality. As a result, investigating novel soil management models to improve the ecological environment of orchards has become a crucial issue that must be resolved for the development and optimization of the jujube industry in semi-arid regions. 

Soil temperature and moisture content are two important habitat factors that influence crop growth and development. Soil moisture levels and distribution can have an effect on soil characteristics, which in turn can have an indirect effect on crop growth [7,8]. Crop growth is greatly influenced by soil temperature, which has a direct effect on root development, water and mineral nutrient uptake, and other metabolic processes [9,10]. Mulching is a crucial component of soil management because it can successfully improve the physical and chemical properties of soil, retain soil moisture, regulate soil temperature, and reduce soil erosion [11,12,13,14,15], all of which contribute to a more favorable environment for crop growth. Filling the water gathering ditch with branches, as suggested by Zhang et al. [16], can improve both water infiltration and the distribution and storage of soil moisture in the vicinity. Mulching with branches has a significant effect on soil water retention during the growing season of fruit trees. Compared to clean tillage, branches mulching increases soil water holding capacity by 10.16% and 10.02%, respectively, at water suctions of 30 and 100 kPa [17]. Grass planted in orchards can reduce soil bulk density, increase soil porosity, and raise the quantity of water-stable aggregates. Moreover, as the number of grass-planting years increases, soil infiltration performance and water-holding capacity increase significantly [18]. The effects of various grass species on soil water storage and its augmentation or diminution are variable [19]. The study also demonstrates that planting grass in an orchard increases surface coverage, regulates soil temperature, prevents the soil temperature from rising rapidly during the summer’s high-temperature period, and contributes to heat preservation at night and during the winter, thereby reducing the annual and daily temperature differences in the soil and enhancing the orchard’s stress tolerance [20]. 

Currently, studies on the effect of mulching on soil moisture and temperature in orchards are primarily conducted on peach, pear, apple, and other types of orchards [21,22,23], while studies on jujube orchards are rarely reported. In particular, there are still insufficient studies on the in-situ mulching and recycling of fruit tree pruning branches. This study utilized branches mulching and grass planting for jujube trees, based on experiments to investigate the performance of clean tillage, jujube branches mulching, and white clover planting on soil moisture and temperature variability, as well as jujube growth and yield. It also proposed appropriate mulching practices in order to provide a scientific foundation for the efficient use of precipitation in rain-fed jujube orchards.

## 2. Results

### 2.1. Effects of Planting Grass and Jujube Branches Mulching on Soil Moisture

#### 2.1.1. Effects of Different Mulching Measures on Annual Variations in Soil Moisture

**Figure 1:**
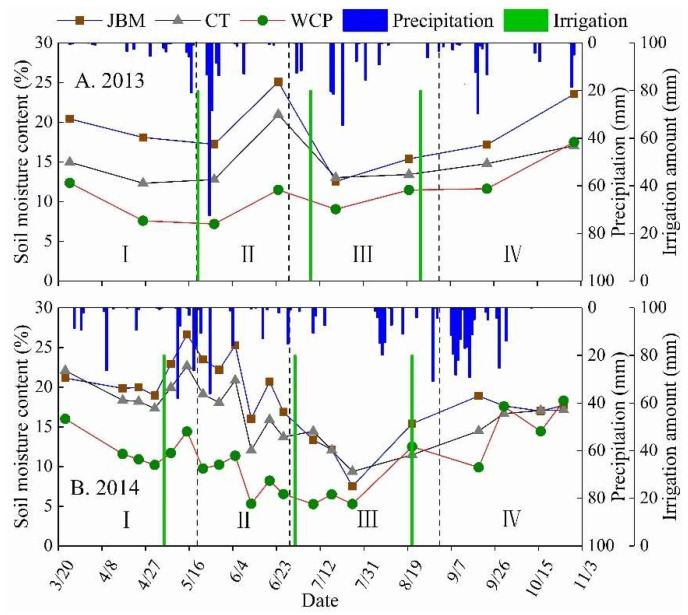
Changes in the 0–70 cm soil layer’s average soil moisture content under different mulching measures during the (**A**) 2013 and (**B**) 2014 jujube growth seasons. JBM: jujube branches were utilized as mulch throughout the entire jujube orchard; CT: clean tillage; and WCP: planting white clover throughout the entire jujube orchard. I: the budding and leaf-expanding stage, II: the flowering and fruit bearing stage, III: the fruit expansion stage, and IV: the fruit ripening stage (similarly hereinafter). Each data point was obtained using the procedure described below: First, average the seven soil moisture measurements of the monitoring point in the 0–70 cm soil layer to represent the soil moisture content of the monitoring point; then, average the soil moisture content of the three monitoring points in the left, center, and right locations to represent the soil moisture content of the soil box; and lastly, average the soil moisture content of the two soil boxes with the same mulching treatment to represent the soil moisture content of the mulching treatment. Due to prolonged periods of no precipitation during the experiment, the soil’s moisture content continued to decline, coming perilously close to wilting moisture. Several irrigations were required to ensure the healthy development of jujube plants and white clover.

The soil moisture content of all three treatments fluctuated dynamically with the occurrence and amount of precipitation during the jujube growing period in 2013 and 2014, and the change processes were comparable (Figure 1). Each treatment’s peak and trough soil moisture content values varied significantly, indicating that mulching practices affected whether soil moisture increased or decreased. Due to a lack of precipitation at this time, the transpiration of jujube trees and soil evaporation led soil moisture to be constantly consumed and caused soil moisture to show varying degrees of loss during the budding and leaf-expanding stage. The amount of precipitation increased significantly, as the budding and leaf-expanding stage began and soil moisture returned. Due to the jujube trees’ aggressive growth during the fruit expansion stage, a significant amount of soil moisture was lost through transpiration. The high air temperature also caused a great deal of soil evaporation, which caused the soil moisture content to drop once more to a low level. As the fruit ripening stage began, the amount of soil moisture lost through transpiration decreased significantly, and soil evaporation decreased steadily. With the additional precipitation, the soil moisture content increased dramatically.

In the 0–70 cm soil layer during the 2013–2014 growing season, JBM had the highest soil moisture content, followed by CT, and WCP had the lowest soil moisture content (Figure 1). JBM had an average soil moisture content of 18.69% during the 2013 jujube growth period, which was 3.76% and 7.65% greater than CT and WCP, respectively (Figure 1A). The difference in soil moisture between different mulching measures was statistically significant (*p* < 0.05). In the growing season of 2014, the average soil moisture content of JBM was 18.68%, which was 2.34% and 7.89% higher than that of CT and WCP, respectively (Figure 1B). Additionally, JBM and CT were significantly different from WCP (*p* < 0.05). Given that jujube branches mulching could effectively improve soil physical properties, increase precipitation infiltration, and block the connection between the atmospheric evaporation layer and surface soil, thereby reducing the ineffective evaporation of soil moisture and improving the soil moisture content, it can be concluded that JBM showed a good capacity to retain soil moisture. During the growing seasons of 2013 and 2014, WCP’s soil moisture content was 3.88% and 5.55% lower than CT (Figure 1). The cause may be that, despite white clover’s extensive coverage of the ground and ability to significantly reduce soil evaporation, transpiration was likely increased due to the plant’s lush growth, which used up more soil moisture than it prevented from evaporating.

#### 2.1.2. Soil Water Deficit under Different Mulching Measures

The term “soil water deficit” refers to a soil whose water content is below its field capacity. Table 1 displays the soil water deficit during jujube development under different mulching measures. Due to frequent and substantial precipitation in May and September, the soil water deficit was reduced in all treatments during the flowering and fruit bearing and fruit ripening stages. The soil water deficit of each treatment was quite severe at other stages. Precipitation was insufficient during the budding and leaf-expanding stage, particularly in 2013, leaving each treatment with a significant soil water deficit. Compared with CT, JBM reduced soil water deficit due to the reduction of soil evaporation by mulching jujube branches. In contrast, white clover entered the squaring stage in WCP when transpiration absorbed a great deal of water, thereby aggravating the soil water deficit. With an increase in precipitation frequency and intensity, the soil water deficit was effectively reduced during the flowering and fruit bearing stage. In particular, the soil moisture in the 20–40 cm soil layer of JBM was fully recovered. In the fruit expansion stage, precipitation decreased, solar radiation was intense, jujube trees transpired vigorously, and soil evaporation intensity was high, resulting in a severe soil water deficit, especially in the 0–20 cm soil layer, indicating that the shallow soil lost a great deal of water during the drought. The soil water deficit of each treatment reversed during the fruit ripening stage in the presence of continuous precipitation.

The average soil water deficit in the 0–70 cm soil layer of JBM was less than that of WCP at different jujube growth stages (Table 1). In 2013’s four stages of jujube growth, the soil water deficit of JBM was successively 17.5%, 36.8%, 2.7%, and 18.5% less than that of WCP. In 2014, the soil water deficit of JBM was less than that of WCP from the budding and leaf-expanding stage to the fruit ripening stage, by 27.7%, 26.4%, 6.7%, and 12.6%, respectively. It can be concluded that the mulching of jujube branches performed better than the planting of white clover in terms of preserving soil moisture. This is primarily due to the fact that mulching jujube branches can combine the promotion of precipitation infiltration and the restriction of soil evaporation, thereby increasing the soil’s water supply in the root zone and maximizing the utilization of precipitation. White clover has a high transpiration rate, which ultimately results in a decrease in soil moisture content and an increase in soil water deficit (Table 1), despite the fact that planting it can increase surface coverage and decrease soil evaporation.

#### 2.1.3. Effects of Planting Grass and Jujube Branches Mulching on the Vertical Distribution of Soil Moisture

In the two experimental years, JBM had the highest annual average soil moisture content in the 0–20 cm soil layer, followed by CT, and WCP had the lowest (Table 2). During the jujube growth period in 2013 and 2014, the average soil moisture of JBM was higher than that of WCP by 3.76% and 3.62%, respectively. It indicates that jujube branches mulching serves the purpose of preserving shallow soil moisture in rain-fed jujube orchards. In the 20–40 cm soil layer, the annual average soil moisture contents of JBM and WCP were 17.69% and 9.11% during the 2013 growth period, and 19.18% and 11.46% during the 2014 growth period. The average annual soil moisture content in CT during the growing seasons of 2013 and 2014 was 15.04% and 17.06%, respectively. While WCP had a lower soil moisture content than CT (*p* < 0.05), we can infer that JBM had a higher soil moisture content than CT. Since 60–65% of white clover roots are dispersed in the top 20–40 cm of soil [24], the moisture content of WCP in this soil layer was low, which is closely related to root water absorption. JBM had the highest annual average soil moisture content in the 40–70 cm soil layer, followed by CT, and WCP had the lowest. It indicates that jujube branches mulching is beneficial to soil moisture conservation in this soil layer. In conclusion, the soil moisture content in the 0–70 cm soil layer of JBM was consistently higher than that of WCP, indicating that mulching with jujube branches was more effective than planting white clover for increasing soil moisture conditions.

### 2.2. Effects of Planting Grass and Jujube Branches Mulching on Soil Temperature

#### 2.2.1. Seasonal Variations in Soil Temperature

**Figure 2:**
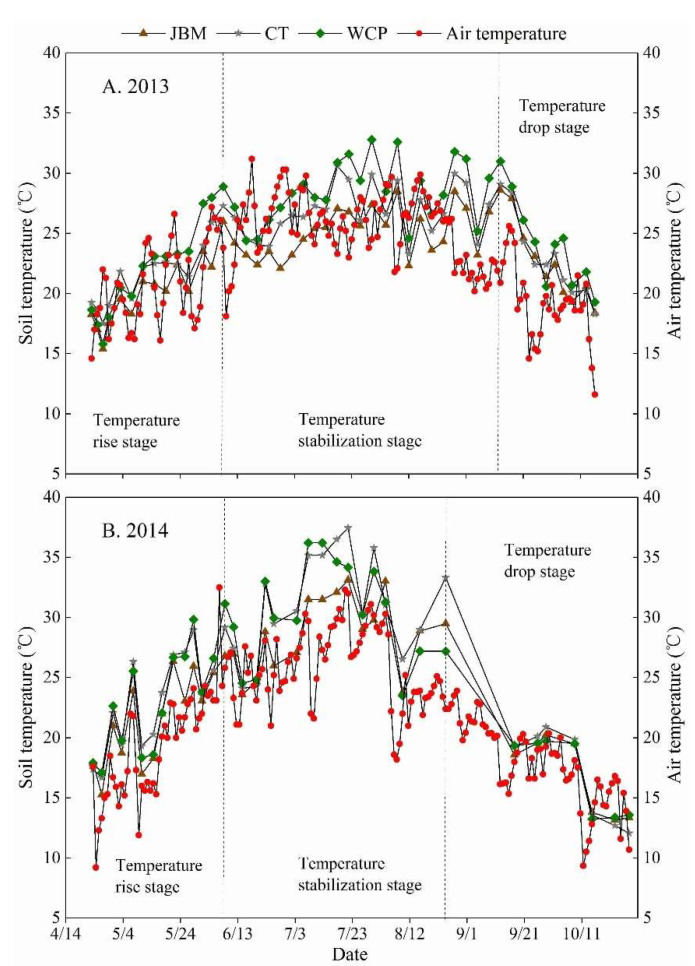
Dynamic changes in the 0–25 cm soil layer’s average soil temperature under different mulching measures over the (**A**) 2013 and (**B**) 2014 jujube growth seasons.

The soil temperature in the 0–25 cm soil layer under all treatments throughout the 2013 and 2014 growing seasons exhibited seasonal variability with the dynamic trend of air temperature, which generally displayed three stages (Figure 2). The temperature rise stage is the first stage (from late April to early June). The increase in solar radiation and rising air temperatures in April caused the surface soil temperature to rise. All treatments’ soil temperatures during this time period showed a general upward trend. Temperature stabilization stage represents the second one (from mid-June to September). Due to the complex and variable external weather conditions during this period, the soil temperature of each treatment varied to a diverse degree, although it was generally stable and maintained at a high temperature. The third stage, known as the temperature drop stage, occurs between September and late October. Throughout this period, as air temperatures decreased and solar radiation diminished, soil temperatures for all treatments showed a downward trend.

In terms of treatments, CT’s soil temperature was the most variable, reaching as high as 17 °C in 2013 and 25.4 °C in 2014 during the growth period (Figure 2). Early on, the soil temperature of the CT treatment rose rapidly, and by mid-May, it was significantly higher than that of the other treatments. The soil’s temperature dropped rapidly after mid-September. Since bare soil was susceptible to environmental influences from the outside, the temperature variation range was quite large. In comparison to CT, the soil temperature of JBM and WCP, which were approximately 13 °C and 20 °C during the growth periods of 2013 and 2014, showed a moderate change, and the seasonal variation range was limited (Figure 2). The soil temperature of JBM and WCP gradually increased during the early stage of the jujube growth period, and then decreased gradually during the late stage. The sluggish heating and cooling of the soil beneath the mulching layer is caused by the virtually static nature of the air in the mulching material and the low thermal conductivity of the air. A significant difference between JBM and CT during the 2013 jujube growing season (*p* < 0.05) indicated that jujube branches mulching had a better ability to regulate soil temperature than clean tillage (Figure 2A).

#### 2.2.2. Diurnal Variation of soil Temperature at Different Depths

**Figure 3:**
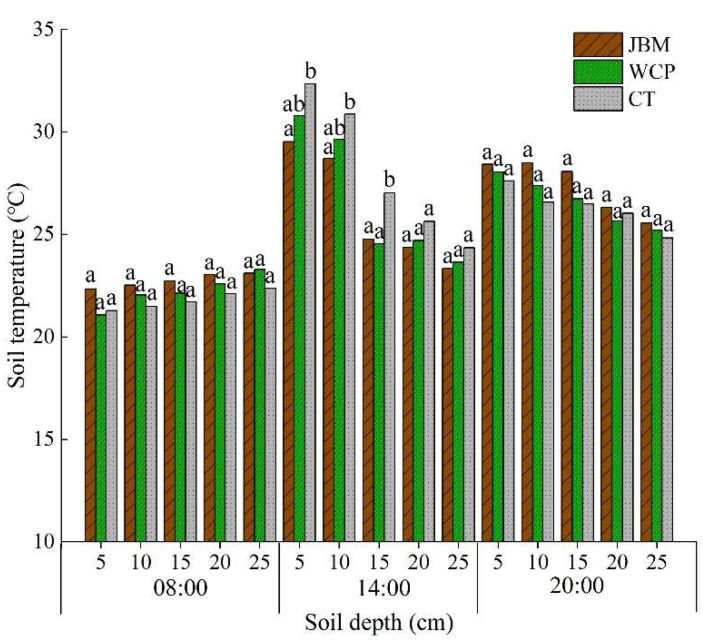
Diurnal variations in soil temperature in the 0–25 cm soil layer at various depths under different mulching measures. At the same time, different lowercase letters at the same depth indicate significant differences in soil temperature for mulching treatments according to Tukey’s test (*p* < 0.05).

Analysis of the same soil layer’s average temperature at the same time in 2014 during the jujube growth period revealed that the temperature increased with increasing soil depth at 08:00 for all treatments, with the highest soil temperature occurring at 25 cm (Figure 3). Each treatment’s soil temperature decreased with increasing depth at 14:00 and 20:00. The lowest soil temperature at each depth of the 0–25 cm soil layer for all treatments appeared at 08:00, the highest soil temperature of the 0–10 cm layer appeared at 14:00, and the highest soil temperature of the 15–25 cm layer appeared at 20:00. The soil temperature of JBM and WCP was marginally higher than that of CT at 08:00 and 20:00, while it was lower at 14:00. It can be concluded that when compared to clean tillage, mulching jujube branches and planting white clover can reduce temperature differences by increasing the temperature of each soil layer in the morning and evening, decreasing the high temperature at noon, improving the diurnal variation of the soil temperature in the shallow layer, and controlling the temperature difference between day and night, which is beneficial for the growth of jujube trees.

#### 2.2.3. Relationship between Soil and Air Temperatures

Using the 2014 jujube growth period as an example, regression analysis was performed on the air temperature, the average temperature of the 0–25 cm soil layer, and the soil temperature at each depth under all treatments to investigate the relationship between soil temperature and air temperature under different mulching measures (Table 3). The regression equation is Y = A + BX, where Y is the soil temperature, X is the air temperature, and A and B are the regression equation’s coefficients.

Under different mulching measures at different depths, there was an extremely strong linear positive correlation between soil temperature and air temperature (*p* < 0.01; Table 3). All correlation coefficients were greater than 0.90, indicating that as the air temperature rose, so did the soil temperature. The average temperature of the 0–25 cm soil layer under CT had the highest response amplitude coefficient (B value), followed by WCP, and the lowest B value under JBM, indicating that CT’s soil temperature was significantly influenced by air temperature while JBM’s soil temperature was less so. The 0–25 cm soil layer showed the highest correlation coefficient between air temperature and soil temperature in CT, followed by WCP, and the lowest correlation coefficient in JBM, indicating that mulching jujube branches and sowing white clover reduced the impact of air temperature on soil temperature. The correlation between soil temperature and air temperature for the same treatment shrank as soil depth increased, indicating that soil temperature was less influenced by air temperature. As soil depth increased, the slope (B value) of the regression equation decreased, demonstrating that the effect of air temperature on soil temperature varied depending on soil depth. In contrast to the deep soil layer, where the soil temperature change was relatively moderate under the influence of air temperature, the topsoil layer’s high B value indicated that the soil temperature changed significantly under the influence of air temperature. 

### 2.3. Effects of Grass Planting and Jujube Branches Mulching on Jujube Tree Growth

LAI is a significant indicator of a plant’s growth health [25]. The LAI changed continuously as the phenological stage of the jujube developed, as shown in Table 4. Under different mulching measures, the LAI variation features of jujube trees were comparable. After germination and leaf expansion, the leaf area grew rapidly until the fruit expansion stage, at which point it reached its maximum. Nonetheless, as the development process progressed, the jujube tree’s vegetative growth slowed, the branches and leaves gradually withered, and the LAI trended downward. The LAI of jujube trees in JBM was significantly greater than in other mulching measures during the same growth period because of favorable soil moisture and temperature conditions, and the average LAI of JBM was 1.3 times that of WCP.

**Figure 4:**
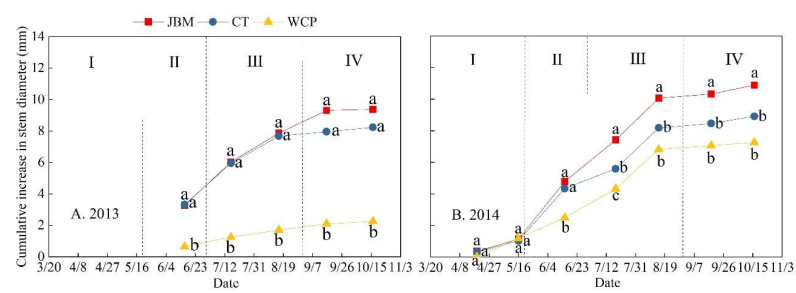
Jujube stem diameter changes over time under different mulching measures in the (**A**) 2013 and (**B**) 2014 jujube growth periods. Different lowercase letters on the same day of the same experimental year indicate significant differences in the cumulative stem diameter growth among treatments, as determined by Tukey’s test (*p* < 0.05). Due to the fact that the 2013 experiment began a bit later than usual, the increase in jujube stem diameter during the budding and leaf-expanding stage could not be measured.

During the two experimental years, the stem diameter of jujube trees increased quickly from the budding and leaf-expanding stage to the fruit expansion stage under all mulching treatments (Figure 4). The tree stem essentially ceased growing after reaching the fruit ripening stage, and its diameter growth tended to be moderate. During the jujube growth period of 2013, JBM had the greatest cumulative increase in jujube stem diameter at 9.38 mm, which was 1.14 and 7.13 mm greater than CT and WCP, respectively, and statistically significant compared to WCP (*p* < 0.05; Figure 4A). In JBM, the growth rate of stem diameter reached 56.8%. The cumulative increase in stem diameter of JBM during the 2014 jujube growth period was 10.89 mm, 1.97 and 3.64 mm greater than that of CT and WCP, respectively (Figure 4B). The growth rate of the stem diameter was 47.7%. The growth of stem diameter in WCP was generally low over the experimental period, particularly during the 2013 jujube growing season, the cumulative increase in stem diameter of WCP was 72.7% less than that of CT (*p* < 0.05; Figure 4A).

## 3. Discussion

Mulching alters the movement and presence of soil moisture, thereby modifying soil moisture content [26,27]. Its primary objective is to alter the physical and chemical properties of the surface and soil. This influences the nature and energy level of soil moisture and water vapor movement [28,29]. JBM demonstrated a high water retention capacity during the experiment. The average soil moisture content of the 0–70 cm soil layer of JBM was 3.76% and 2.34% higher than that of CT during the jujube growth season in 2013 and 2014 (Figure 1). The explanation is that jujube branches mulching creates an aquifer on the ground that improves soil infiltration and prevents soil moisture from evaporating into the atmosphere. Two processes contribute to soil evaporation under surface mulching: water loss from the mulching layer and soil evaporation beneath the mulching layer. In the second process, soil moisture is prevented from escaping into the atmosphere by the mulching layer, and cumulative soil evaporation beneath the mulching layer is low compared to bare soil without mulching [30,31,32]. It is advantageous to improve soil water utilization efficiency in arid regions. 

The water deficit in each soil layer from 0 to 70 cm was significant in various precipitation years, and the soil moisture content of WCP was significantly lower than that of CT (Figure 1 and Table 1). This phenomenon may be attributable to the fact that white clover grows abundantly and requires a great deal of soil water due to its high transpiration rate. This contradicts the findings of Li et al. [33] and Hernández et al. [34] that grass cultivation in orchards can increase soil moisture content. For instance, orchards in southern China receive an average of more than 800 mm of precipitation annually, and orchards in certain developed countries may be irrigated or receive a great deal of local precipitation [35,36,37,38]. The effect of planting grass on water retention in orchards is somewhat different, as this study focuses primarily on arid and semi-arid rain-fed agricultural environments. According to Li et al. [39], grass cultivation in orchards typically leads to competition between grass and fruit trees for soil moisture in regions with low precipitation. Water and fertilizer competition exists between grass and fruit trees, especially in the spring and summer, and the negative impact on fruit trees is exacerbated in dry years. Therefore, the ecological suitability of planting grass in orchards is an issue that merits consideration when promoting grass planting in rain-fed orchards. Particularly in the soil management of rain-fed jujube orchards with low annual precipitation, care should be taken to plant grass.

Compared to CT, JBM and WCP demonstrated soil temperature regulation effects in the 0–25 cm soil layer. Specifically, the seasonal variation range of soil temperature and the difference between daytime and nighttime soil temperatures throughout the jujube development period were reduced (Figure 2 and Figure 3). JBM had the greatest effect on regulating soil temperature and was least affected by air temperature (Table 3). Numerous studies have demonstrated that mulching has a thermal insulation effect when the external environment is cold and can effectively prevent the soil temperature from rising extremely quickly when the external environment is hot, all of which can help to maintain the soil temperature at a relatively stable and favorable state for crop root growth [40,41,42,43]. Growing grass in an orchard increases surface coverage, reduces direct solar exposure during hot months, slows heat transmission to the deep soil, and gradually raises the surface soil temperature. Grass can help keep the soil warm during chilly nights, thereby decreasing the soil’s daily temperature difference and increasing the orchard’s cold tolerance. Mulching with jujube branches is a type of organic material mulching that differs from mulching with inorganic materials such as plastic film, which is mostly dependent on temperature increase. Its temperature regulation effect is as follows: when the air temperature is high, it has a cooling effect; when the air temperature is low, it has a heat preservation effect, thereby enhancing the soil’s ability to withstand sudden fluctuations in air temperature. The authors discovered that the study area’s air temperature rose rapidly in the spring, and that jujube branches mulching could significantly slow the soil temperature’s ascent. In the summer, the soil’s maximum temperature was kept at a low level by the mulching of jujube branches, which was beneficial for the jujube trees’ root metabolism.

This study discovered that the leaf area index and stem diameter growth of jujube under JBM were significantly greater than under other mulching measures (Table 4 and Figure 4). This may be because jujube branches mulching has a good ability to regulate soil moisture and soil temperature, thereby enhancing the environment for root development and promoting jujube growth, which is consistent with the findings of numerous studies [44,45,46]. The jujube branches used in the experiment originated from pruned jujube trees. In addition to achieving the triple objectives of preserving soil moisture, regulating soil temperature, and encouraging the growth of jujube trees, the pruned jujube branches were used to mulch in situ, saving mulching materials and realizing the recycling of agricultural waste, which provides scientific support for the development of sustainable ecological agriculture and water-saving agriculture.

## 4. Conclusions

The soil water deficit in the 0–70 cm soil layer was minimal under JBM, and soil moisture content increased by 3.76% and 2.34% on average during the jujube growth period in 2013 and 2014, respectively, when compared to CT. The soil water deficit was severe under WCP, and the average soil moisture content during the jujube growth period in 2013 and 2014 was 3.88% and 5.55% lower than that of CT, respectively. JBM had the highest annual average soil moisture content, followed by CT, while WCP had the lowest in each soil layer from 0 to 70 cm. White clover and jujube competed for water in the 20–40 cm soil layer of WCP, whereas JBM had the least variation in soil moisture. Both JBM and WCP were able to adjust the soil temperature in the 0–25 cm soil layer and limit the diurnal range of soil temperature. JBM’s soil temperature was also the least impacted by air temperature. Jujube stem diameter growth and leaf area index in JBM were both significantly higher than those in CT and WCP. In conclusion, jujube branches mulching is a suitable practice for rain-fed jujube orchards because it can preserve soil moisture, regulate soil temperature, and promote the growth of jujube plants.

## 5. Materials and Methods

### 5.1. Overview of the Study Area

From March 2013 to October 2014, the experiment was conducted at the experimental base of Northwest A&F University’s Institute of Water-saving Agriculture in Arid Areas (34°18′ N, 108°04′ E, 521 m a. s. l.), in the Yangling Demonstration Zone, Shaanxi Province, China. The annual average air temperature in this region is 13 °C, with an average variation between day and night of 11.5 °C. The number of days without frost each year ranges from 169 to 200. The annual average precipitation is 635.5 mm, and the seasonal distribution is irregular, with 60% of the precipitation falling between July and October. Since the study area received minimal precipitation from November to March of the following year, the total precipitation during the jujube growth period (April to October) served as the precipitation measurement for the experimental year in this study. The year 2013 was deemed a dry year due to the fact that the total precipitation during the jujube growth period was only 448.8 mm, a decrease of more than 10% from the annual average precipitation [47]. The year 2014 was regarded as a normal precipitation year because the total precipitation during the jujube growth period was 588.5 mm, a decrease of less than 10% from the annual average precipitation [47]. 

### 5.2. Experimental Design

**Figure 5:**
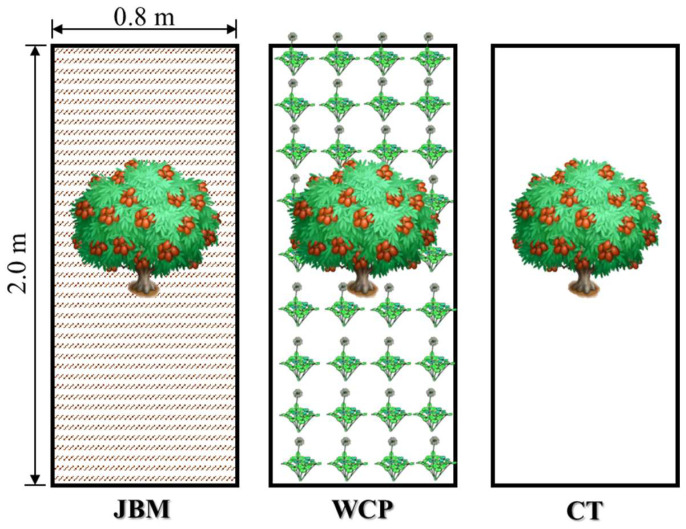
Layout diagram for experimental treatments (JBM, WCP, and CT).

The experiments of several mulching measures served as the foundation for this study. All experimental plots were made of soil boxes with dimensions of 2.0 m in length, 0.8 m in width, and 0.8 m in height. The soil boxes had transparent plexiglass on both sides and tiny holes evenly spaced at the bottom to maintain a balanced air pressure that permitted soil moisture to freely permeate. Three experimental treatments were established in this study: jujube branches were mulched throughout the orchard (JBM), white clover (*Trifolium repens* L.) was planted throughout the orchard (WCP), and clean tillage (CT) served as the control (Figure 5). Each mulching treatment was repeated twice. In this study, there were consequently six experimental plots, or six soil boxes. Prior to the formal experiment, when the white clover began to turn green in early March, the soil moisture content (soil volumetric moisture content, similarly referred to hereafter) of the 0–70 cm soil layer in each experimental plot was measured and the water storage capacity was estimated. This was done to ensure that the initial soil moisture content of each plot was identical. Using the plot with the highest soil water storage capacity as the benchmark, quantitative supplemental irrigation was applied to the remaining plots with lower soil water storage capacities. A watering can is used for hand-held spraying to ensure uniform irrigation. Since the soil box was placed on the ground, white foam boards were embedded and attached on both sides of the soil box to achieve the goal of heat insulation and prevent solar radiation and air temperature from affecting soil temperature and evaporation.

### 5.3. Materials for Experiments

The Lizao jujube variety, with an average initial height of 24.5 cm, was planted in the experimental plot on 20 November 2009. Each soil box contained a single jujube tree. The annual growth of jujube trees is divided into four stages in this study based on experimental observations and records: (I) the budding and leaf-expanding stage (early April to mid-May), (II) the flowering and fruit bearing stage (late May to late June), (III) the fruit expansion stage (early July to late August), and (IV) the fruit ripening stage (early September to late October). On 5 March 2011, white clover was sown at a density of 15 g∙m^−2^. The jujube branches used in the experiment were pruned from jujube trees, which were chopped to a length of approximately 8 cm, and then scattered across the soil’s surface at a thickness of 10 cm. Loessial soil with a mass water content of approximately 5% was placed in the soil box after being processed via a 10 mm sieve and moderately air-dried naturally. According to field soil bulk density data collected by Gao et al. [48], the soil dry density is maintained between 1.35 and 1.40 g∙cm^−3^. Beginning with the bottom of the soil box, seven 10 cm-thick soil layers were added. In order to reduce the negative effect of stratification between soil layers on water infiltration, the soil was evenly compacted and the surface of the preceding soil layer was roughened before the next layer was added. The soil’s fundamental physical characteristics are presented in Table 5.

### 5.4. Observation Indicators and Methods

#### 5.4.1. Soil Moisture

**Figure 6:**
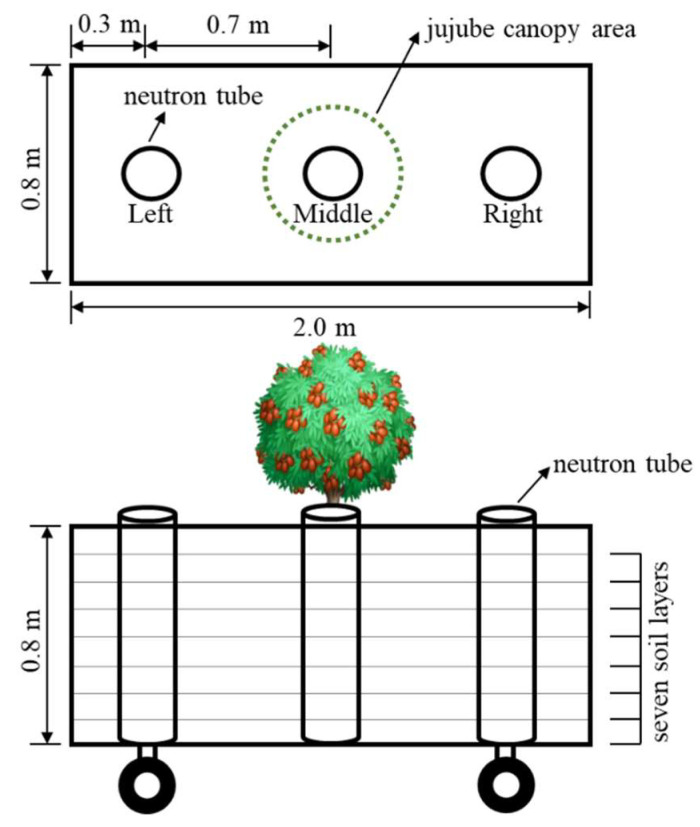
Layout diagram of neutron tubes in experimental plots.

The soil volumetric moisture content was measured using a CS830 neutron probe (Nanjing Chishun Science and Technology Co., Nanjing, China). Each experimental plot’s left, middle, and right positions were used to collect soil moisture (Figure 6). A layer was defined as every 10 cm in each location, and the average of three measurements was used to determine the soil moisture content of each layer. 

#### 5.4.2. Soil Temperature

In each experimental plot, a set of geothermometers was installed in two-thirds of the canopy projection of jujube trees. The observation depths were 5, 10, 15, 20, and 25 cm. The soil temperature was measured at 08:00, 14:00, and 20:00 every three days.

#### 5.4.3. Jujube Canopy Leaf Area Index

The leaf area index (LAI) was measured using an AccuPAR LP-80 plant canopy analyzer (Decagon Devices Inc., Pullman, WA, USA). On a sunny day, measurements were taken at four different locations (east, south, west, and north) on the jujube tree between 10 a.m. and 2 p.m. The average of these measurements served as the LAI of the jujube tree in the corresponding plot.

#### 5.4.4. Jujube Stem Diameter

Prior to the experiment, a circle was drawn with a marker pen 10 cm above the ground on the trunk of the jujube tree. The diameter of the trunk was then measured in the east-west and south-north directions using a vernier caliper with an accuracy of 0.01 mm, and the mean value was used as the stem diameter baseline. As the jujube tree grew, the stem diameter was measured in the middle of each month.

#### 5.4.5. Meteorological Data

Yangling National Meteorological Observing Station, located approximately 100 m from the experimental site, provided precipitation and air temperature for this investigation.

### 5.5. Data Processing

Soil volumetric moisture content was calculated as follows [49]:

*θ_v_* = *a* + *b* (*cnt/std*) (1)

where *θ_v_* is the soil volumetric moisture content, %; *a* and *b* are the intercept and slope of the calibration equation, respectively; *cnt* is the original data collected by the neutron probe in the soil; *std* is the standard count of the neutron probe under standard indoor conditions.

Soil water storage was calculated according to the following formula:

*W* = 10*Hθ_v_* (2)

where *W* is the amount of stored water in the soil, mm; *H* is the thickness of the soil layer, cm.

The soil water deficit was determined as follows:

*D* = (*F* − *W_c_*)/*F* × 100% (3)

where *D* is the soil water deficit, %; *F* is the field capacity, mm; *W_c_* is the actual soil water storage, mm. *D* > 0 denotes the presence of a soil water deficit, and a larger *D* denotes a more severe one. *D* = 0 denotes the absence of a soil water deficit.

### 5.6. Statistical Analysis

One-way analysis of variance (ANOVA) with Tukey’s post-hoc test was performed to separate means at a significance level of *p* < 0.05.

## Figures and Tables

**Table 1:** Soil water deficit in the 0–70 cm soil layer at each growth stage of jujube under different mulching measures.

Experimental Year	Soil Layer/cm	Mulching Measures	Soil Water Deficit (%)			
			Budding and Leaf-Expanding Stage	Flowering and Fruit Bearing Stage	Fruit Expansion Stage	Fruit Ripening Stage
2013	0–20	JBM	38.1a	8.5a	52.8a	23.0a
		CT	47.8b	30.1a	53.5b	32.9b
		WCP	53.2b	44.8b	51.3a	39.7a
	20–40	JBM	20.6a	0.0a	30.9a	4.2a
		CT	34.3b	13.4b	37.4a	13.3a
		WCP	48.1c	43.2c	42.7b	39.5b
	40–70	JBM	32.1a	1.4a	41.7a	14.3a
		CT	39.4b	19.9b	40.0a	21.2b
		WCP	42.0c	32.3c	39.6b	17.7c
	Mean	JBM	30.2a	3.3a	41.8a	13.8a
		CT	40.5b	21.1b	43.7a	22.5b
		WCP	47.8c	40.1c	44.5b	32.3c
2014	0–20	JBM	20.3a	4.8a	47.2a	17.6a
		CT	23.4a	20.5a	59.6a	21.2a
		WCP	45.7b	37.3b	61.2a	23.6a
	20–40	JBM	2.0a	0.0a	39.5a	2.7a
		CT	8.6a	7.3a	42.9a	7.4a
		WCP	39.3b	29.1b	43.6a	21.9a
	40–70	JBM	13.5a	0.0a	38.0a	2.4a
		CT	13.4a	7.7b	41.6ab	9.4a
		WCP	33.9b	17.7c	40.2b	15.0a
	Mean	JBM	11.9a	1.6a	41.6a	7.6a
		CT	15.1a	11.9a	48.0a	12.7a
		WCP	39.6b	28.0b	48.3a	20.1a

Note: Different lowercase letters in the same soil layer at the same growth stage during the same experimental year show significant differences in soil water deficit across mulching treatments, as determined by Tukey’s test (*p* < 0.05).

**Table 2:** Statistical properties of the vertical distribution of soil moisture in the 0–70 cm soil layer under different mulching measures.

Soil Layer/cm	Mulching Measures	Average Soil Moisture/%	
		2013	2014
0–20	JBM	12.74a	14.64a
	CT	12.24a	14.41a
	WCP	8.98a	11.02a
20–40	JBM	17.69a	19.18a
	CT	15.04a	17.06a
	WCP	9.11b	11.46b
40–70	JBM	17.20a	18.73a
	CT	14.01ab	16.36ab
	WCP	10.54b	12.76b

Note: Those without the same lowercase letter in the same soil layer during the same experimental year indicate that the difference in average soil moisture reaches a significant level of 5% based on Tukey’s test (*p* < 0.05).

**Table 3:** Regression relationship between air and soil temperatures at different depths under different mulching measures.

Mulching Measures	Soil Depth/cm	Regression Coefficient		Correlation Coefficient
		A	B	
JBM	5	2.987	1.000	0.960 **
	10	0.831	1.014	0.960 **
	15	1.158	1.001	0.951 **
	20	2.986	1.002	0.937 **
	25	1.807	0.994	0.901 **
	Mean	1.954	0.988	0.965 **
CT	5	1.306	1.226	0.966 **
	10	0.906	1.198	0.964 **
	15	−2.517	1.181	0.958 **
	20	0.674	1.121	0.958 **
	25	0.193	1.077	0.953 **
	Mean	0.112	1.161	0.949 **
WCP	5	0.621	1.198	0.964 **
	10	0.199	1.124	0.959 **
	15	0.080	1.108	0.957 **
	20	1.885	1.093	0.946 **
	25	−0.040	1.078	0.910 **
	Mean	0.549	1.120	0.956 **

Note: ** represents that the correlation coefficient reaches an extremely significant level (*p* < 0.01).

**Table 4:** Changes in LAI at different growth stages under different mulching measures during the 2014 jujube growing season.

Mulching Measures	LAI			
	Budding and Leaf-Expanding Stage	Flowering and Fruit Bearing Stage	Fruit Expansion Stage	Fruit Ripening Stage
JBM	0.67a	0.93a	1.33a	1.16a
CT	0.57a	0.86a	1.29a	1.13a
WCP	0.41a	0.67a	1.14a	1.11a

Note: The different lowercase letters at the same growth stage indicate that there is significant difference in LAI among treatments based on Tukey’s test (*p* < 0.05).

**Table 5:** Physical properties of experimental soil.

Particle Size/mm	Content/%	Saturated Hydraulic Conductivity/(mm∙min^−1^)	Saturated Water Content/%	Field Capacity/%	Wilting Moisture/%
0.02–2	17.6 ± 1.3	0.49 ± 0.15	53.7 ± 1.9	29.4 ± 0.96	8.44 ± 1.32
0.002–0.02	64.3 ± 1.8				
<0.002	18.1 ± 2.6				

## Data Availability

Data is contained within the article.
